# Impact of Education and Experience on Radiographers’ Ability to Diagnose Acute Appendicitis: A Survey in Private Malaysian Hospitals

**DOI:** 10.21315/mjms2024.31.2.16

**Published:** 2024-04-23

**Authors:** M. L. Dinesh, Mohd Imran Mohd, B. R. Shasindrau, Daniel Jeyaraman

**Affiliations:** Department of Medical Imaging, KPJ Healthcare University, Negeri Sembilan, Malaysia

**Keywords:** radiographer’s knowledge, acute appendicitis, CT scan, MRI, ultrasound scan

## Abstract

**Background:**

Acute appendicitis is a global surgical emergency. Radiographic modalities usually identify acute appendicitis, although radiographers’ competence is questionable. This study examines how clinical radiographers’ education and experience affect their ability to identify acute appendicitis using computed tomography (CT), magnetic resonance imaging (MRI) and ultrasonography (USG) characteristics. The study also aimed to determine which variable strongly influences their knowledge level.

**Methods:**

The study surveyed radiographers with a four-part self-administered questionnaire containing demographic information and eight knowledge-based questions about the appearance of acute appendicitis in MRI, CT and USG, separately. Before distribution, the questionnaire was validated and checked the reliability.

**Results:**

Clinical radiographers’ knowledge about using MRI to diagnose acute appendicitis was strongly affected by education and experience (η^2^ = 0.13 and 0.14; *P* < 0.05), with bachelor’s degree holders scoring higher regardless of experience. Radiographers with more than 5 years of experience knew more about CT and USG features to identify acute appendicitis (η^2^ = 0.40 and 0.27; *P* < 0.05). Radiographers with a bachelor’s degree and greater experience had higher overall knowledge of MRI, CT and USG to diagnose acute appendicitis (η^2^ = 0.51 and 0.11; *P* < 0.05). With adjusted *R*^2^ = 54% (*F* [2, 44] = 27.94; *P* < 0.001), education and experience highly predicted the overall knowledge level.

**Conclusion:**

The study found gaps in radiographers’ knowledge of the radiographic appearance of acute appendicitis. Clinical radiographers’ education level and years of experience substantially affect their knowledge level. In addition, experience is a good predictor than education level for overall knowledge level. Therefore, the study emphasises the importance of continuing education and training for radiographers to diagnose acute appendicitis quickly and accurately.

## Introduction

Acute appendicitis is one of the most common reasons for urgent surgery and can affect people of any age, with a 20.5% worldwide increase between 1990 and 2019 and a 7% lifetime incidence (1, 2). Radiography plays an integral part in the diagnosis of acute appendicitis. Computed tomography (CT) and ultrasonography (USG) are often used to diagnose acute appendicitis (3). Radiographers use their knowledge of acute appendicitis’ radiographic appearance to diagnose the condition quickly and accurately. Correct radiographic interpretation can help diagnose acute appendicitis. These include distention, wall thickening and peri-appendiceal inflammation (3).

Radiographers produce high-quality radiographs and help diagnose medical conditions (4). They work closely with radiologists and other medical specialists to diagnose various illnesses accurately and quickly, including acute appendicitis. Ofori-Manteaw and Dzidzornu (5) found that radiographers and junior physicians could improve their accuracy in detecting radiographic abnormalities and commenting on trauma radiographs with training. However, radiographers’ radiographic knowledge of specific diseases is unclear. It is a significant concern because radiographers make the basic radiographic interpretation.

Radiographers’ skills in interpreting acute appendicitis images are poorly understood, despite the importance of radiography in diagnosing acute appendicitis. Many studies have examined radiographic imaging’s accuracy and diagnostic performance in acute appendicitis assessment (6–8). However, none addressed radiographers’ radiographic interpretation skills of any diseases, including acute appendicitis. According to an Australian study by Baird (9), radiographers need to improve their image interpretation of all pathologies, and that study also suggests that radiographers’ training in pathology diagnosis, including acute appendicitis, may be lacking.

This study aims to determine the influence of clinical radiographers’ education level and years of working experience on their overall knowledge level to diagnose acute appendicitis by the radiographic characteristics in CT scan, magnetic resonance imaging (MRI) and ultrasound imaging modalities. The study also aimed to identify which variable strongly influences knowledge level in this area. This study used a survey among radiographers as the primary data collection method. The findings of this study will provide insights into the current level of knowledge that radiographers possess and identify any potential gaps in their knowledge regarding the radiographic appearance of acute appendicitis and its impact on patient outcomes.

## Methods

### Participants

Clinical radiographers from various private hospitals in Malaysia were chosen for the study based on their availability and willingness to take part. The Krejcie and Morgan’s (10) table was used to calculate the sample size and 47 participants were recruited using convenience sampling techniques. The inclusion criteria were that all the participants should be certified clinical radiographers with at least 1 year of experience and use radiographic modalities to diagnose acute appendicitis.

### Research Design

A cross-sectional survey design was used based on the study’s aims and objectives. This method is appropriate for gathering information on radiographers’ current knowledge and experience with the radiographic appearance of acute appendicitis and collects data at a particular point in time, allowing for a snapshot of the participant’s knowledge and expertise. In addition, it can examine how radiographers’ education and experience affect their knowledge and skill, allowing a complete and cost-effective study of the research question.

### Data Collection Process

The data collection procedure included the distribution of a four-part self-administered questionnaire. The first section of the questionnaire gathered demographic information such as age, gender, education and work experience. The second section of the questionnaire included eight knowledge-based questions about the MRI scan appearance of acute appendicitis and the third section included eight knowledge-based questions about the CT scan appearance of acute appendicitis. Finally, the fourth section of the questionnaire included eight knowledge-based questions about the appearance of acute appendicitis on an ultrasound scan.

The questionnaire’s validity and reliability were checked before sending it out. First, the questionnaire’s face and content validity were determined by a panel of experts consisting of three medical imaging lecturers and three senior radiographers. The questionnaire was changed based on their suggestions and comments. The Cronbach’s alpha coefficient was used to measure the questionnaire’s reliability and was found to be 0.86, which means that the questionnaire has a high internal consistency and reliability (11).

The questionnaire was then delivered to the participants who met the inclusion criteria and data was collected over 4 weeks. The study’s objective was explained to the participants and their informed consent was obtained before their participation. Participants were also told that their participation in the study was completely voluntary and that their responses would be kept strictly confidential. Participants were told to complete the questionnaire independently and return it to the researcher within the stipulated time.

### Statistical Analysis

Data collected from the questionnaire were entered into Microsoft Excel v.16.70 and then into the Statistical Package for the Social Sciences (SPSS) version 26.0, ensuring the data was in the proper format and all variables were correctly labelled. Next, the data was cleaned and pre-processed to identify missing values, outliers or errors. This step involved recoding and transforming some variables, such as converting text to numeric data or recoding categorical variables, ensuring the data was suitable for analysis. In this study, descriptive and inferential statistics such as two-way ANOVA and multiple linear regression (MLR) were utilised in the data analysis.

## Results

A The sample was characterised using descriptive statistics. Table 1 shows 47 participants with valid data for all variables. Twenty participants, comprising 42.6% of the sample, had 1 year–5 years of experience, while 27 participants, constituting 57.4% of the sample, had over 5 years of experience. Most participants (*n* = 29, 61.7%) had a radiography diploma, while 18 (38.3%) had a bachelor’s degree. Females outnumbered males (59.6%, *n* = 28 versus 40.4%, *n* = 19). Knowledge level of MRI (KLMRI) scores ranged from 21 to 33 out of 40, with a mean of 26.23 (standard deviation [SD] 3.04). Knowledge level of CT (KLCT) scores were 20–35 out of 40, with a mean of 27.60 (SD 3.20). The 40-point knowledge level of the USG (KLUSG) scale ranged from 18 to 34, with a mean of 25.87 (SD 4.39). Finally, the overall knowledge level (OKL) scores ranged from 65 to 99 out of 120, with a mean of 79.70 (SD 8.06).

### Effects of Education Level and Year of Experience on KLMRI Scores

KLMRI’s means, SDs and *F*-ratio are presented in Table 2. The results showed significant mean differences in KLMRI based on education level (*F* [1, 43] = 6.38, MSE = 7.15, *P* = 0.015, η^2^ = 0.13) and year of experience (*F* [1, 43] = 6.79, MSE = 7.15, *P* = 0.013, η^2^ = 0.14) but the effect size was medium (0.06 ≤ η^2^ < 0.14) (12). However, *F* (1, 43) = 0.011, MSE = 7.15, *P* = 0.917 and η^2^ = 0.00 show no significant mean difference between the product of education level and years of experience with KLMRI. An in-depth analysis showed that bachelor’s degree holders with 1 year–5 years of experience had higher mean KLMRI scores (M = 26.33, SD 1.63) than diploma holders (M = 24.14, SD 2.14). Bachelors (M = 28.42, SD 3.5) had higher mean KLMRI scores than diploma holders (M = 26.40, SD 2.67) with > 5 years of experience.

### Effects of Education Level and Year of Experience on KLCT Scores

Table 2 summarises KLCT means, SDs and *F*-ratio. The results showed that KLCT scores differed significantly by experience (*F* [1, 43] = 28.69, MSE = 6.44, *P* < 0.001, η^2^ = 0.40) and had a large effect size (η^2^ ≥ 0.14). The means do not differ significantly based on education level (*F* [1, 43] = 0.35, MSE = 6.44, *P* = 0.557, η^2^ = 0.01) or ‘year of experience multiplied by the level of education’ (*F* [1, 43] = 0.43, MSE = 6.44, *P* = 0.518, η^2^ = 0.01). The mean KLCT values for those with 1 year–5 years of experience were similar for those with a bachelor’s degree and those with a diploma (M = 25.17, SD 2.14 and M = 25.21, SD 2.67, respectively). Bachelor’s degree holders with more than 5 years of experience had higher mean KLCT scores (M = 29.92, SD 2.31) than diploma holders (M = 28.93, SD 2.71).

### Effects of Education Level and Year of Experience on KLUSG Scores

KLUSG’s means, SDs and *F*-ratio are depicted in Table 2. *F* (1, 43) = 15.86, MSE = 14.00, *P* < 0.001, η^2^ = 0.27, having a large effect size (η^2^ ≥ 0.14), showed significant mean differences in KLUSG by years of experience. However, there is no significant mean difference between education level (*F* [1, 43] = 1.53, MSE = 14.00, *P* = 0.223, η^2^ = 0.03) and the product of education level and years of experience (*F* (1, 43) = 0.44, MSE = 14.00, *P* = 0.510, η^2^ = 0.01). In the 1-to-5-year experience range, the bachelor’s degree group and the diploma group have nearly identical mean KLUSG values (M = 23.67, SD 3.27 and M = 23.00, SD 3.49, respectively). However, in the group with more than 5 years of experience, bachelor’s degree holders (M = 29.08, SD 3.53) had higher mean KLUSG scores than diploma holders (M = 26.87, SD 4.26).

### Effects of Education Level and Year of Experience on OKL Scores

Means, SDs and the *F*-ratio for OKL are provided in Table 2. The results showed that there were significant mean differences in OKL based on the number of years of experience with *F* (1, 43) = 41.56, MSE = 30.26, *P* < 0.001, η^2^ =.51 having a large effect size (η^2^ ≥ 0.14) and on education level with *F* (1, 43) = 5.49, MSE = 30.26, *P* = 0.024, η^2^ = 0.11, but with a medium effect size (0.06 ≤ η^2^ < 0.14). Though with *F* (1, 43) = 0.49, MSE = 30.26, *P* = 0.486 and η^2^ = 0.01, there is no evidence of a significant mean difference between OKL and the product of education level and years of experience. In the 1 year–5 years of experience group, those with a bachelor’s degree (M = 75.17, SD 5.91) had slightly higher mean OKL scores than those with a diploma (M = 72.36, SD 4.43). In the group of people with more than 5 years of experience, those with a bachelor’s degree (M = 87.42, SD 6.1) again showed slightly higher mean OKL scores than those with a diploma (M = 82.20, SD 5.77).

### Association between OKL with Education Level and Years of Experience

**Figure 1:**
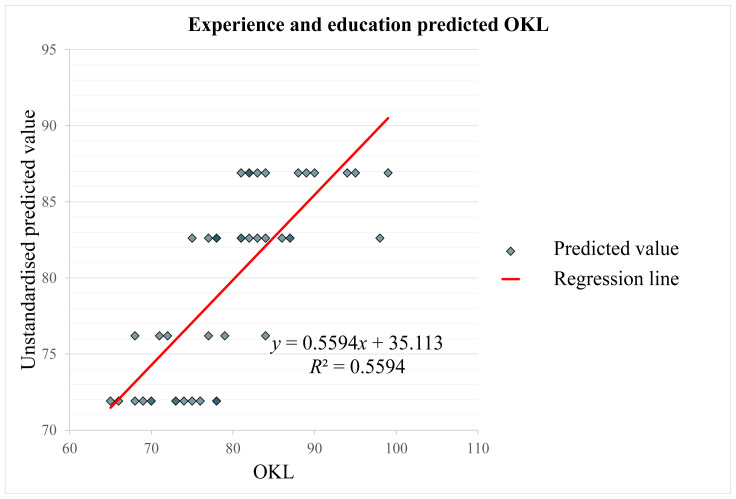
Scatter plot with regression line showing education level and years of experience predicting OKL

Multiple regression analysis illustrates the OKL’s association with education and experience in Table 3. Linearity, homoscedasticity, normality and absence of multicollinearity assumptions were fulfilled. Figure 1 shows that the model explains 56% of the variance in OKL with adjusted *R*^2^ = 54% and is statistically significant (*F* [2, 44] = 27.94, *P* < 0.001) (13). The clinical radiographers’ knowledge level significantly grows by 10.70 units for every year of experience, *t* (44) = 6.56, *P* < 0.001. However, a one-unit increase in education level improves the OKL by 4.29 units, which was similarly significant, *t* (44) = 2.58, *P* = 0.013. The effect of years of experience was larger (*β* = 0.663) than education level (*β* = 0.261). Table 4 shows that OKL was positively correlated with education (*r* = 0.36, *P* = 0.013) and experience (*r* = 0.70, *P* < 0.001), but years of experience and education had no significant correlation (*r* = 0.147, *P* = 0.324). According to standardised beta coefficients, years of experience predicted OKL better than education. These findings imply that clinical radiographers with more working experience and higher academic degrees are better at using radiographic modalities to identify acute appendicitis, with working experience being the strongest predictor.

## Discussion

Our findings suggest that education level and years of experience influence KLMRI with medium effect sizes (0.13 and 0.14, respectively). Previous research has found that education and radiography clinical knowledge correlate positively (14, 15). Clinical radiographers with bachelor’s degrees showed higher mean KLMRI scores than those with diplomas in the 1–5 and 5+ year experience groups. It suggests that more education may improve the MRI scan diagnosis of acute appendicitis. Alsharif et al. (16) observed comparable results in their Saudi Arabian clinical radiographers’ MRI knowledge study.

This study found that clinical radiographers’ knowledge of using CT and USG images to identify acute appendicitis differed significantly by experience, *P* < 0.001. It supports previous studies showing that experience affects radiographers’ understanding (17, 18). Clinical radiographers with more than 5 years of experience may have higher mean KLCT and KLUSG scores because they are more familiar with their equipment and technology. Our investigation found no significant variation in mean KLCT and KLUSG scores by education level (*P* > 0.05). It contradicts previous research. Such studies showed that clinical radiographers’ competence in using CT and USG scans to diagnose medical conditions grew dramatically with education (19, 20). Our study only used CT or USG scans to diagnose acute appendicitis, which may explain the discrepancies.

The bachelor’s degree and diploma groups in the 1 year–5 years of experience category had identical KLCT (M = 25.17, SD 2.14 and M = 25.21, SD 2.67, respectively) and KLUSG (M = 23.67, SD 3.27 and M = 23.00, SD 3.49, respectively) mean values. It suggests that clinical radiographers with fewer than 5 years of experience do not benefit from formal education in using CT or USG scans to diagnose acute appendicitis. This study may have been too small a sample size to find a connection between KLCT and KLUSG and educational level. Nevertheless, this study suggests that clinical radiographers’ experience may affect their understanding of CT and USG scans in acute appendicitis diagnosis. Bachelor’s degrees do not boost knowledge depth compared to diploma holders. So, clinical radiographers must get ongoing education and training to keep up with imaging technologies and preserve their skills.

This study also examines how clinical radiographers’ education and experience predict their OKL. Multiple regression analysis showed that education and experience were significant predictors (*R*^2^ = 54%) of overall knowledge. However, the working experience was shown to be a substantially higher effect size (*β* = 0.663, *P* < 0.001) than education level (*β* = 0.261, *P* = 0.013) in predicting OKL. Clinical radiographers learn imaging modalities best through experience, according to the study. These findings match the current study. Al-Dahery et al. (21) observed that experienced clinical radiographers knew more about using MRI for accurate diagnosis. Similarly, Murphy et al. (22) and van de Venter and ten Ham-Baloyi (23) also discovered that radiographers with more education and experience performed and interpreted radiographic images better.

This review found that the present radiography education system is ineffective. Those with over 5 years of work experience know more, regardless of their education level. However, persons with less than 5 years of work experience are equally knowledgeable regardless of education. Higher education should mean having good knowledge, but the study indicates otherwise. Doesn’t that reflect a failing education system? This study focused on clinical radiographers’ knowledge of using MRI, CT or USG scans to identify acute appendicitis, although it was hypothesised that they would have approximately the same knowledge about other disorders’ radiographic appearance. Radiographers in most hospitals have less than five years of experience. Their ignorance will lower the radiology department’s quality. So, the quality and education level of radiographers ought to be increased.

Future research should consider the study’s limitations. First, the study did not assess clinical radiographers’ knowledge and skills in diagnosing acute appendicitis. Thus, future research could examine how education and experience affect clinical radiographers’ practical skills. The present study examined clinical radiographers’ knowledge of only MRI, CT and USG scans for acute appendicitis diagnosis. Future research may examine other diagnostic imaging methods or medical conditions. Finally, the sample size was small and the study was limited to one region. Future studies should replicate these findings in multiple regions to ensure generalisability and consider a larger sample size to improve external validity.

## Conclusion

This research aimed to determine the degree of expertise of clinical radiographers in using MRI, CT and USG imaging modalities to diagnose acute appendicitis. The study also examined the effect of education level and years of experience on clinical radiographers’ total level of knowledge. The findings revealed that both education level and years of experience substantially affect the knowledge level of clinical radiographers. The results suggested that clinical radiographers with a bachelor’s degree had a higher level of knowledge than those with a diploma qualification. In addition, the results revealed that clinical radiographers with more years of experience were more proficient than those with less experience.

The effect of experience was found to significantly impact the knowledge level of clinical radiographers regarding the usage of all three types of scans. However, clinical radiographers’ education level did not significantly predict their knowledge level in diagnosing acute appendicitis. According to the study’s findings, a clinical radiographer’s overall knowledge level can be significantly predicted by their education level and years of experience. On the other hand, it was observed that the impact of years of experience was significantly greater than the impact of the level of education.

The study found that experienced clinical radiographers better diagnose acute appendicitis with MRI, CT and USG scans, and years of experience predict OKL better than education. These findings have significant implications for clinical radiographers’ training and progress. So, clinical radiographers should renew their knowledge of how CT, USG and MRI are used to diagnose acute appendicitis and other illnesses through ongoing education and training. This research may provide valuable insights into radiographer education and training, which could lead to a more well-rounded and efficient educational programme for pitch advancement. In addition, hospitals should hire clinical radiographers with advanced degrees for the best patient outcome.

## Figures and Tables

**Table 1:** Descriptive statistics for year of experience, education, gender, KLMRI, KLCT, KLUSG and OKL

Demographic variables				Test variables		

	Category	*n* (%)		Range	Mean	SD
			
Experience	1–5 years	20 (42.6)				
	> 5 years	27 (57.4)	KLMRI	21–33	26.23	3.04
Education	Diploma	29 (61.7)	KLCT	20–35	27.60	3.24
	Degree	18 (38.3)	KLUSG	18–34	25.87	4.39
Gender	Male	19 (40.4)	OKL	65–99	79.70	8.06
	Female	28 (59.6)				

Notes: *n* = 47; SD = standard deviation; *n* = sample size for each category; KLMRI = clinical radiographers’ knowledge level to diagnose acute appendicitis using MRI; KLCT = clinical radiographers’ knowledge level to diagnose acute appendicitis using CT; KLUSG = clinical radiographers’ knowledge level to diagnose acute appendicitis using USG; OKL = clinical radiographers’ overall knowledge level to diagnose acute appendicitis using MRI, CT and USG

**Table 2:** Means, SDs and two-way ANOVA statistics for clinical radiographers’ knowledge level about using MRI, CT, USG and OKL to diagnose acute appendicitis

Variable	Diploma		Bachelor		ANOVA			

	Mean	SD	Mean	SD	Effect	*F*-ratio	df	η^2^
	
KLMRI								
1–5 EXP	24.14	2.14	26.33	1.63	ED	6.38**	1,43	.13
> 5 EXP	26.40	2.67	28.42	3.50	EXP	6.79**	1,43	.14
					ED × EXP	0.011	1,43	.00
KLCT								
1–5 EXP	25.21	2.67	25.17	2.14	ED	0.35	1,43	.01
> 5 EXP	28.93	2.71	29.92	2.31	EXP	28.69***	1,43	.40
					ED × EXP	0.43	1,43	.01
KLUSG								
1–5 EXP	23.00	3.49	23.67	3.27	ED	1.53	1,43	.03
> 5 EXP	26.87	4.26	29.08	3.53	EXP	15.86***	1,43	.27
					ED × EXP	0.44	1,43	.01
OKL								
1–5 EXP	72.36	4.43	75.17	5.91	ED	5.49**	1,43	.11
> 5 EXP	82.20	5.77	87.42	6.10	EXP	41.56***	1,43	.50
					ED × EXP		1,43	.01

Notes: *n* = 47; SD = standard deviation; ANOVA = analysis of variance; ED = education level; EXP = year of experience;

** *P* < 0.05;

*** *P* < 0.001

**Table 3:** Linear regression analysis summary for education and experience predicting OKL (*n* = 47)

Factors	Adjusted B (95% CI)^a^	*β*	*t*	*P-value* b
(Constant)	56.93 [50.32, 63.54]		17.35	< 0.001
Experience	10.70 [7.41, 14.00]	0.66	6.56	< 0.001
Education	4.29 [0.94, 7.63]	0.26	2.58	0.013

Notes:

a Adjusted regression coefficients; CI = confidence interval for B;

b Multiple linear regression (*R*^2^ adjusted = 0.54)

**Table 4:** Correlation between OKL, education level and years of experience (*n* = 47)

Variables	Correlation coefficient (*r*)	*P*-value
OKL - years of experience	0.702	< 0.001
OKL - education level	0.359	0.013
Years of experience - education level	0.147	0.324

Note: OKL = clinical radiographers’ overall knowledge level to diagnose acute appendicitis using MRI, CT and USG
